# Ether in Boston

**Published:** 1849-11

**Authors:** 


*Ether in Boston.*—The following sentence occurs in the advertisement of the Massachusetts Medical College:—"The safe and effectual practice of etherization is taught in this school."—*Western Lancet.*

